# The World Health Organization's work and recommendations for improving the health of trans and gender diverse people

**DOI:** 10.1002/jia2.26004

**Published:** 2022-10-12

**Authors:** Virginia Macdonald, Annette Verster, Maeve B. Mello, Karel Blondeel, Avni Amin, Niklas Luhmann, Rachel Baggaley, Meg Doherty

**Affiliations:** ^1^ Global Programme for HIV Hepatitis and STIs World Health Organization Geneva Switzerland; ^2^ UNDP‐UNFPA‐UNICEF‐WHO‐World Bank Special Programme of Research, Development and Research Training in Human Reproduction (HRP), Department of Sexual and Reproductive Health and Research World Health Organization Geneva Switzerland

**Keywords:** transgender, HIV, World Health Organization, policy, guideline, key populations

## Abstract

**Introduction:**

The World Health Organization (WHO) is guided by its global programme of work and the goal that a billion more people have universal health coverage (UHC). To achieve UHC, access for those most vulnerable must be guaranteed and prioritized. WHO is committed to developing evidence‐based guidance to work towards UHC for trans and gender diverse (TGD) people. This commentary describes WHO's work related to TGD people over the last decade.

**Discussion:**

In 2011, WHO developed guidelines for the prevention and treatment of HIV and sexually transmitted infections (STIs) in men who have sex with men and TGD people. In 2013, the “HIV civil society reference group” called on WHO to provide specific guidance for TGD people. Values and preferences of TGD people were considered by WHO for the first time, which informed the development of the 2014 WHO Consolidated Guidelines on HIV Prevention, Diagnosis, Treatment and Care for Key Populations. The 2014 Guidelines included a comprehensive package of HIV‐related health and enabling interventions with specific considerations for TGD people, as well as a specific policy brief in 2015. Regional WHO offices developed and/or supported the development of blueprints on transgender health and HIV in 2014 and 2016. A 2015 WHO report on sexual health, human rights and the law elucidated the harmful impacts of discriminatory laws on the basis of sexual orientation and gender identity. In 2019, the 11th edition of the international classification of diseases saw the removal of “transsexualism” as a mental and behavioural disorder. WHO's first guideline on self‐care interventions, updated in 2021, included key considerations concerning TGD people. In 2022, WHO's updated key populations guidelines include a prioritized package of not just HIV, but also viral hepatitis and STI health interventions for TGD people. Still, a broader and more specific health approach and a greater focus on social issues are needed to better serve the health needs of TGD people.

**Conclusions:**

WHO's understanding and commitment to TGD people's health has evolved and improved over the past decade. Together with professional and community trans health organizations, WHO should now start developing evidence‐informed global guidance on TGD health as part of its remit to support UHC to all.

## 1 INTRODUCTION

The Thirteenth General Programme of Work defines the World Health Organization's (WHO's) strategy for 2019–2023. It focuses on triple billion targets to achieve measurable impacts on people's health at the country level. The triple billion targets are to ensure by 2023 that 1 billion more people are better protected from health emergencies; 1 billion more people are enjoying better health and wellbeing and 1 billion more people are benefiting from universal health coverage (UHC) (1). UHC means that all people can use the promotive, preventive, curative, rehabilitative and palliative health services and commodities they need, of sufficient quality to be effective, while also ensuring that the use of these services does not expose the user to financial hardship (2).

For trans and gender diverse (TGD) people, as well as other key populations for the HIV epidemic, there are additional barriers to reaching UHC. Structural barriers which limit access to health services for TGD people include stigma, discrimination, violence, criminalization and lack of legal gender recognition. These can be compounded by other vulnerabilities, such as disability, ethnicity, migrant status, sexual orientation and poverty. In countries where TGD people are officially recognized, health insurance schemes rarely cover specialized care for this community, including gender‐affirming care. Further, the absence of trans knowledgeable and trained clinicians who can provide quality, specialized care can deter TGD people from accessing health services (3). But the concept of UHC, in which “no one is left behind” and the most vulnerable should be prioritized (progressive universalism), provides opportunities for TGD people to advocate for the inclusion of their specific health needs in national health packages (4). As WHO supports countries to realize UHC, some countries are also working towards better representation of TGD people in policy and planning and a better understanding of and guidance on how to address their specific health needs (5). In this commentary, we look back at WHO's development of norms and standards for TGD people's health and wellbeing.

## 2 DISCUSSION

Historically, within WHO, there has been limited focus on the healthcare needs of TGD people. Only in 2008, WHO held a global consultation on “Prevention and treatment of HIV and other sexually transmitted infections (STI) for men who have sex with men and transgender populations,” and highlighted for the first time that “[…] many transgender people object to being labelled as men who have sex with men, since they do not identify themselves as men.” (6). This statement considered only transgender women, excluding transgender men who have sex with men. Recommendations from the 2008 global consultation called for WHO to develop guidance for delivering an evidence‐informed package of interventions to prevent and treat HIV and STIs among transgender women and men who have sex with men. It is important to note that the term “men who have sex with men” was adopted by epidemiologists, focusing exclusively on sexual behaviours which lead to HIV acquisition and transmission rather than cultures, communities and identities, considerably overlooking gender diversity and population‐specific health needs. Further, the focus at this stage was only on transgender women due to their high burden of HIV infection with insufficient evidence on the burden of HIV infection in transgender men. In 2011, the WHO HIV department developed such guidelines and, while recognizing that transgender women were a separate population from men who have sex with men, no transgender‐specific recommendations were made (7). Rather, all recommendations concerning men who have sex with men were indirectly applied to transgender women.

In 2013, the HIV civil society reference group, an advisory group to the WHO, called on WHO to provide specific recommendations for TGD people, recognizing that this was a continuing major gap in WHO guidance. In response, a qualitative assessment of TGD people's values and preferences related to HIV was commissioned by WHO, the first global WHO study that explicitly included trans men. Results showed that there was poor availability of trans‐specific health information and persistent barriers to access and utilization of health services, including stigma, discrimination, legal constraints related to gender recognition, criminalization and violence. Results also showed a lack of understanding and training among health workers to provide gender‐sensitive and gender‐affirming care and particularly that TGD people prioritized gender‐affirming care over other health interventions, including those related to STIs and HIV (8).

The Pan American Health Office, WHO regional office for the Americas (PAHO/WHO), had also flagged TGD health as an issue to which WHO headquarters needed to provide better global guidance. Aware of the need to bring visibility to the trans community in the English‐speaking Caribbean, PAHO/WHO published the “Blueprint for the Provision of Comprehensive Care for Trans Persons and their Communities in the Caribbean and other Anglophone Countries” in 2014 (9). The “Blueprint” was published after PAHO/WHO's Member States approved a resolution for countries to address the causes of disparities in health service access and utilization for lesbian, gay, bisexual and trans (LGBT) persons (10), which was followed, in 2018, by a report analysing their situation in the Americas (11). The report overtly recommended countries to “cease to regard transgender identities as pathology,” “post visible non‐discrimination statements that explicitly refer to sexual orientation and gender identity/expression, and visitation rights for same‐sex/‐gender partners in cases of hospitalization,” “collect qualitative and quantitative data on sexual orientation and gender identity to monitor any obstacles that LGBT people face when accessing health services and barriers,” among others. In 2015, following a similar format, the Asia Pacific region released the “Blueprint for the Provision of Comprehensive Care for Trans People and Trans Communities” led by the United Nations Development Programme (UNDP) in close partnership with WHO and others (12).

The regional Blueprints provide a practical guide to help countries implement health services for TGD people. Both include how to conduct respectful reception and registration of TGD people and physical exam describing in detail the steps to providing TGD‐sensitive care. Furthermore, they provided guidance on specific healthcare related to body modification, including hormone therapy and surgery for gender affirmation. Additionally, in 2016, WHO worked with other United Nations (UN) partners, technical agencies and networks of TGD people to develop the TRANSIT, an implementing tool for comprehensive HIV and STI Programmes with Transgender People, which focused on supporting and empowering TGD communities (13).

WHO headquarters’ role within WHO is to set norms and standards. In 2014, the WHO published the “Consolidated Guidelines on HIV Prevention, Diagnosis, Treatment and Care for Key Populations” promoting a comprehensive package for HIV‐related interventions for key populations. This guidance and related policy brief included specific recommendations for TGD people, such as those related to access to sterile needles and syringes for injecting hormones, cervical cancer screening for transgender men, use of oral contraceptives and interactions between hormones for gender affirmation and antiretroviral drugs. The guideline also included language for better quality and sensitive healthcare provision for TGD people recommending that healthcare providers should be sensitive to and knowledgeable about the specific health needs of TGD people, in particular during the genital examination and specimen collection. This was the first time that TGD‐specific health interventions were included in a WHO guideline (8, 14).

The 2014 Consolidated Guidelines were also the first global guidance to include enabling interventions as part of a suggested comprehensive package of interventions for HIV in response to the impact that structural barriers have on TGD people and other key populations’ access and utilization of health services (8, 14).

One year later, the specific needs of young people who are TGD were addressed in a WHO technical brief, which highlighted the complexities of addressing HIV while recognizing and realizing different gender identities among youth, the possible accompanying mental health issues, potential stigma and isolation from families and communities (15). Additionally, in 2016, the WHO recommended pre‐exposure prophylaxis for all people at substantial risk of HIV, including transgender women (16).

In 2015, WHO was a signatory to a joint statement with other UN agencies calling for an end to discrimination and violence against gender and sexual minorities, including in healthcare settings (17). Also, in 2015, WHO published a report on sexual health, human rights and the law, elucidating the harmful impacts of discriminatory laws on the basis of sexual orientation and gender identity and recommending ways to improve services, including information on gender‐affirming care, gender‐sensitive healthcare services and addressing violence against TGD people (18). In 2016, the Joint United Nations Programme on HIV/AIDS (UNAIDS) and the WHO Global Health Workforce Alliance jointly launched the Agenda for Zero Discrimination in Health Care (19). The agenda set out a seven‐piece action plan, all relevant for eliminating stigma against TGD people, which included removing legal and policy barriers, setting standards for discrimination‐free healthcare, community empowerment, and mechanisms and frameworks for monitoring, evaluation and accountability.

Ongoing analysis of the adoption of the recommendations included in the 2016 HIV key populations consolidated guidelines in the WHO African region show consistently low uptake of those specific to TGD people, with only 10 out of 49 (20%) National HIV Strategic Plans (NSP) from the region including this population in 2020. A similar analysis in the Americas region demonstrated a greater uptake, but still with less than half (6 out of 14) of the Caribbean countries having referred to trans or gender diverse people in their NSPs. On the other hand, 100% (19) of Latin American countries cited trans or gender diverse people in their NSP and 58% of them included language on the need to review national laws, policies or practices that criminalize trans or gender diverse people. Additionally, 42% of NSPs from Latin American countries recommended the training of health providers to be sensitive to this population.

As part of its normative role, WHO is also responsible for the International Classification of Diseases (ICD) and works on its development with several stakeholders, including communities. The ICD is used to define eligibility and access to health services and health insurance and can facilitate the collection of data that guides policy and programme decisions. From 1992 to 2019, the ICD classified variations of the binomial male‐female as “transsexualism” as a mental and behavioural disorder (20). This classification reinforced stigma and barriers to care for TGD people. For example, under the ICD‐10 classification, many TGD people required a diagnosis from a psychiatrist before they were able to access gender‐affirming care where available. Further, the previous classification created an environment where mental health issues, such as depression and anxiety, were misdiagnosed and poorly managed in TGD people. The TGD communities advocated to remove “transsexualism” from the ICD and after an extensive review of the evidence, in 2019 the ICD‐11 replaced the prior classification with the concept of “gender incongruence” and defined it as a condition within the sexual health chapter rather than as a mental and behavioural disorder (21). Some advocates called for the removal of gender identity from the ICD‐11 altogether, given its classification as an issue of sexual health is also inaccurate (22). However, inclusion in the ICD helps to ensure TGD people's access to gender‐affirming healthcare as well as health insurance coverage. This change should reduce barriers and move TGD people one step closer to equity in health coverage, in countries where they are recognized.

Given the paucity of evidence about trans health beyond HIV (23) and in diverse geographies and legal contexts, evidence‐informed recommendations for TGD people using WHO standards (24) can be difficult to make. The gaps in evidence and knowledge of the health needs of TGD populations call for specific research with attention to safe and ethical methods to include them in research and develop inclusive data collection systems (25). In 2019, WHO developed Consolidated Guidelines on Self‐care Interventions for Health: Sexual and Reproductive Health and Rights, which were updated in 2021 (26). For the first time, these guidelines contain key considerations related to the self‐administration of gender‐affirming hormones for TGD people. The guidelines development group urgently called for more research to support further evidence‐informed guidance for TGD people in order to support a WHO recommendation.

In 2022, the 2016 WHO Consolidated Key Population Guidelines have been updated and include recommendations related to viral hepatitis and STI prevention, diagnosis, treatment and care for TGD and other key populations alongside HIV‐related recommendations (27). For this update, WHO commissioned values and preferences research through the TGD people's global network GATE (Global Action for Trans Equality) to inform the development of the guidelines. The guidelines also include prioritized packages of health interventions for each key population group, highlighting, in the case of the TGD people, the importance of national programmes establishing and providing gender‐affirming care or effective linkage and referral to services which can provide such care. As in prior editions, the Consolidated Guidelines make the case for recognition of TGD peoples’ gender identity in official documents to improve access to healthcare.

## 3 CONCLUSIONS

WHO's role, to promote health for all, means it is committed to inclusive healthcare and equitable access for trans, gender diverse and all other people. In December 2020, Dr Tedros Adhamon Ghebreyesus, Director‐General of WHO tweeted “Ultimately, our fight is not against a single disease. Our fight is against a world in which people get sick and die simply because they are poor, or female, or young, gay, transgender, sex workers, use drugs or are in prison. Our fight is for #HealthForAll” (28), demonstrating WHO's highest level commitment to support countries to attend to the health needs of TGD people. While commitment from WHO and other global partners, including donors is crucial, ultimately it is for countries to make the necessary changes, to recognize gender diversity and remove structural barriers to ensure UHC which also includes TGD people. WHO's understanding and commitment to TGD people's health and wellbeing has evolved and improved, but there is still further to go. While WHO has demonstrated increasing commitment to engaging meaningfully, and on an equal basis with TGD communities, this partnership should be further strengthened. Together with professional and community trans health organizations, such as GATE, WPATH and other UN cosponsors, WHO should facilitate more research and start developing evidence‐informed global guidance to improve TGD people's health beyond HIV, viral hepatitis and STIs as part of its remit to support UHC to all.

## COMPETING INTERESTS

No competing interests exist.

## AUTHORS’ CONTRIBUTIONS

AV and VM wrote the main text and coordinated input. KB contributed considerable text as well. MBM, RB, NL, AA and MD reviewed the text and provided additional text.

## FUNDING

This work received funding from the UNDP‐UNFPA‐UNICEF‐WHO‐World Bank Special Programme of Research, Development and Research Training in Human Reproduction (HRP), a cosponsored programme executed by the World Health Organization (WHO) and the Bill and Melinda Gates Foundation.

## Data Availability

Authors alone are responsible for the views expressed in this publication and do not necessarily represent the decisions or the policies of the UNDP‐UNFPA‐UNICEF‐WHO‐World Bank Special Programme of Research, Development and Research Training in Human Reproduction (HRP) or the World Health Organization (WHO).
